# Ovarian cancer symptoms in pre-clinical invasive epithelial ovarian cancer – An exploratory analysis nested within the UK Collaborative Trial of Ovarian Cancer Screening (UKCTOCS)

**DOI:** 10.1016/j.ygyno.2023.11.005

**Published:** 2023-11-18

**Authors:** James Dilley, Aleksandra Gentry-Maharaj, Andy Ryan, Matthew Burnell, Ranjit Manchanda, Jatinderpal Kalsi, Naveena Singh, Robert Woolas, Aarti Sharma, Karin Williamson, Tim Mould, Lesley Fallowfield, Stuart Campbell, Steven J. Skates, Alistair McGuire, Mahesh Parmar, Ian Jacobs, Usha Menon

**Affiliations:** a Department of Gynaecological Oncology, Barts Health NHS Trust, London, UK; b MRC Clinical Trials Unit at UCL, Institute of Clinical Trials & Methodology, University College London, London, UK; c Department of Women’s Cancer, Elizabeth Garrett Anderson Institute for Women’s Health, University College London, London, UK; d Wolfson Institute of Population Health, CRUK Barts Cancer Centre, Queen Mary University of London, London, UK; e Department of Cellular Pathology, Barts Health NHS Trust, London, UK; f Department of Gynaecological Oncology, Queen Alexandra Hospital, Portsmouth, UK; g Department of Obstetrics and Gynaecology, University Hospital of Wales, Cardiff, UK; h Department of Gynaecological Oncology, Nottingham University Hospitals, Nottingham, UK; i Department of Gynaecological Oncology, University College London Hospitals NHS Trust, London, UK; j Sussex Health Outcomes Research and Education in Cancer (SHORE-C), Brighton and Sussex Medical School, University of Sussex, Sussex, UK; k Create Health London, London, UK; l Massachusetts General Hospital and Harvard Medical School, Harvard, MA, USA; m London School of Economics, London, UK

**Keywords:** Ovarian cancer, Symptoms, UKCTOCS, GOFF index, NICE

## Abstract

**Objective:**

UKCTOCS provides an opportunity to explore symptoms in preclinical invasive epithelial ovarian cancer (iEOC). We report on symptoms in women with pre-clinical (screen-detected) cancers (PC) compared to clinically diagnosed (CD) cancers.

**Methods:**

In UKCTOCS, 202638 postmenopausal women, aged 50–74 were randomly allocated (April 17, 2001-September 29, 2005) 2:1:1 to no screening or annual screening till Dec 31,2011, using a multimodal or ultrasound strategy. Follow-up was through national registries. An outcomes committee adjudicated on OC diagnosis, histotype, stage. Eligible women were those diagnosed with iEOC at primary censorship (Dec 31, 2014). Symptom details were extracted from trial clinical-assessment forms and medical records. Descriptive statistics were used to compare symptoms in PC versus CD women with early (I/II) and advanced (III/IV/unable to stage) stage high-grade-serous (HGSC) cancer. ISRCTN-22488978; ClinicalTrials.gov-NCT00058032.

**Results:**

1133 (286PC; 847CD) women developed iEOC. Median age (years) at diagnosis was earlier in PC compared to CD (66.8PC, 68.7CD, *p* = 0.0001) group. In the PC group, 48% (112/234; 90%, 660/730CD) reported symptoms when questioned. Half PC (50%, 13/26PC; 36%, 29/80CD; *p* = 0.213) women with symptomatic HGSC had >1symptom, with abdominal symptoms most common, both in early (62%, 16/26, PC; 53% 42/80, CD; *p* = 0.421) and advanced (57%, 49/86, PC; 74%, 431/580, CD; *p* = 0.001) stages. In symptomatic early-stage HGSC, compared to CD, PC women reported more gastrointestinal (change in bowel habits and dyspepsia) (35%, 9/26PC; 9%, 7/80CD; p = 0.001) and systemic (mostly lethargy/tiredness) (27%, 7/26PC; 9%, 7/80CD; *p* = 0.017) symptoms.

**Conclusions:**

Our findings, add to the growing evidence, that we should reconsider what constitutes alert symptoms for early tubo-ovarian cancer. We need a more nuanced complex of key symptoms which is then evaluated and refined in a prospective trial.

## Graphical abstract

**Graphical abstract:**
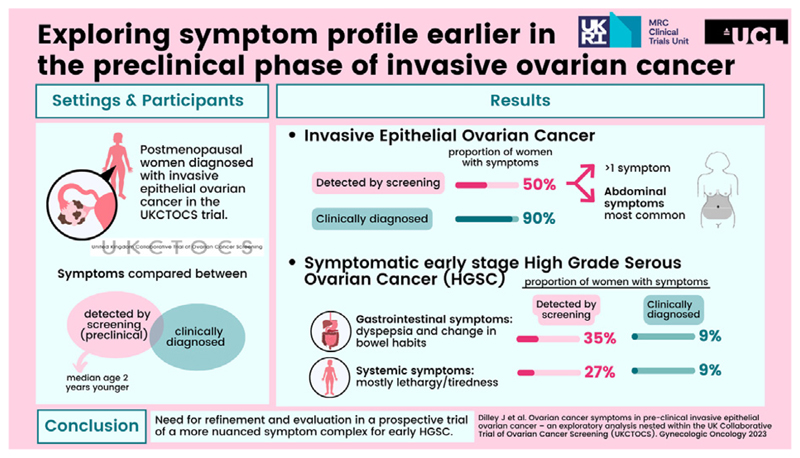

## 1 Introduction

Ovarian and tubal cancer remain the most lethal of all gynaecological malignancies as a majority of women are diagnosed with aggressive advanced stage high-grade serous tubo-ovarian cancers (HGSC). Early-stage cancers have a much-improved survival [1]. However, efforts at early detection in the UK Collaborative Trial of Ovarian Cancer Screening (UKCTOCS) did not result in a reduction in disease specific mortality [2,3]. Consequently, diagnosis based on symptom recognition remains the only currently available approach.

In the UK and US widespread OC national guidelines [4,5] and awareness campaigns recommend that postmenopausal women seek medical advice and specialist referral if they have any ‘high alert’ symptoms. However, it has become apparent that these symptoms do not facilitate detection at an earlier stage. Women with these symptoms have poorer survival [6]. The high alert symptoms were informed by insights gained over the past 20 years from women clinically diagnosed with ovarian cancer [7–9]. There is increasing awareness that high grade serous tubo-ovarian cancers (HGSC) spend on average >4 years as in situ / early stage and approximately 1 year as stage III or IV cancers before becoming clinically apparent [10]. To date we have limited insights on the symptoms that women might experience during this preclinical phase. The downstaging [2,3] with improved treatment outcomes in women with HGSC in the multimodal (MMS) screening group of UKCTOCS [11], provides an opportunity to explore for the first time symptoms in women detected with ovarian cancer earlier in its natural history, prior to clinical diagnosis.

We report the symptom profiles of women with cancers detected by screening in the screened arms of UKCTOCS and compare these data with symptoms reported by those clinically diagnosed in the control and screen arms of the trial. Insights gained would contribute to refining the symptom constellation of ovarian cancer ‘high alert’ symptoms.

## 2 Materials and methods

The randomised controlled trial, UKCTOCS, was designed to answer whether population screening would improve detection and therefore impact on ovarian cancer mortality. It was approved by the UK North-West MREC (00/8/34) on June 23, 2000. All women provided written consent. The trial design has been previously published [2,3] and the protocol is available online [12].

In brief, following random invitation of 1,243,282 women from population registers of 27 Primary Care Trusts adjoining 13 trial centres in England, Wales and Northern Ireland, 202,638 women were recruited and randomised between April 17, 2001 and September 29, 2005. Inclusion criteria were age 50–74 years and postmenopausal status. Exclusion criteria were bilateral oophorectomy, previous ovarian or active non-ovarian malignancy or increased familial OC risk. Gender was initially based on NHS age-sex register information and then self-confirmed at recruitment as at least one intact ovary was an eligibility criterion. Ethnicity and other baseline characteristics were self-reported at recruitment.

The trial management system confirmed eligibility and then randomly allocated women using the Visual Basic randomisation statement and the Rnd function to no screening (Control group – 101,359) or annual screening using a multimodal (MMS 50,640) or ultrasound (USS, 50,639) strategy. It allocated 32 random numbers to each trial centre, of which eight were allocated to MMS, eight to USS and the remaining 16 to no screening. We randomly allocated each successive participant within the centre to one of the numbers and subsequently randomly allocated them into a group. Investigators and participants were aware, and the outcomes committee was masked to randomisation group.

Women in the MMS and USS groups underwent a median of 8 (range 7–11) annual screens between 17 April 2001 and 31 December 2011. In both groups, women with persistent abnormalities on screening underwent clinical assessment by trial clinicians using a trial specific clinical assessment form that included questions probing symptoms. If suspicious, the participants were referred to the NHS for further investigation and trial surgery. We deemed women who had surgery or a biopsy for suspected ovarian cancer after clinical assessment as screen positive. Screen-detected cancers were those diagnosed following positive screen findings.

### 2.1 Follow-up and confirmation of diagnosis

Participants were followed up via electronic health record linkage to national cancer and death registrations [13] and to hospital episode statistics. Additional sources included two rounds of postal questionnaires (3–5 years after randomisation and in 2014) and direct communication from participants. Censorship date for this analysis was 31 Dec 2014. As previously detailed, medical notes were retrieved for all women with notification of a possible ovarian or tubal cancer diagnosis. An independent outcomes review committee, masked to randomisation group, assigned the final diagnosis, date of diagnosis, FIGO 2014 stage, histotype and cause of death (where applicable).

### 2.2 Subjects

All women with confirmed diagnosis of invasive epithelial ovarian and tubal cancer on outcome review between randomisation and censorship for primary outcome (Dec 31, 2014) were included in the current analyses [3]. Women with non-epithelial and borderline epithelial tumours were excluded.

Women were grouped based on screening status into (1) those with pre-clinical disease (PC) - women with screen detected cancers diagnosed following positive results on screening in the MMS and USS groups (2) clinically diagnosed (CD) – women with all other iEOC in the MMS, USS and no screening group.

### 2.3 Symptom ascertainment and classification

Symptom data was retrieved by a single clinician (JD). Data sources included, hospital notes, multidisciplinary gynaecological oncology team summaries and copies of the primary care physician (general practitioner) and hospital letters. In addition, symptom data was extracted from the trial clinical assessment form for PC women. All reported symptoms with onset in the ≤12 months preceding diagnosis were captured. Longstanding symptoms defined as those persisting >12 months were excluded. No limit was placed on the number of symptoms that could be recorded for each woman.

Women were classified as ‘symptomatic’ if they had reported any symptoms or ‘asymptomatic’ if this was documented or no symptoms were mentioned despite the availability of comprehensive documentation. Women with ‘insufficient’ documentation were classified as having missing data. The symptoms were grouped both by modified Goff Symptom index (GSI) which included abdominal or pelvic pain, increased abdominal size or bloating and loss of appetite/feeling full. The original Goff symptom index includes duration and frequency of symptoms. As frequency was often not captured in the hospital notes, we were unable to include it in our analysis. We also grouped by National Institute for Health and Care Excellence (NICE) UK guidance on ovarian cancer symptoms (NSG) [4] which in addition to the above symptoms included increased urinary urgency or frequency. Symptoms were also grouped according to system (gynaecological, abdominal, gastrointestinal, urinary, systemic, other) as detailed in eTable1 and described previously [6,9]. Symptoms not previously described were allocated to the most appropriate system upon agreement of two clinical researchers (JD and UM).

### 2.4 Statistical analysis

Baseline characteristics of the eligible women in the PC and CD groups were calculated. For this exploratory analysis, descriptive statistics including tabulations were calculated for proportion of women with symptoms, positive GSI and positive NSG. In symptomatic women, median number of symptoms and proportions with positive symptoms in the various systems (gynaecological, abdominal, gastrointestinal, urinary, systemic, other) were calculated.

We explored if there were differences in the symptom profile of PC women compared to CD women

1. Overall
2. By histotype and stage defined as

Histotype - (1) High-grade (grade 2–3) serous tubo-ovarian carcinoma (HGSC) using grade and histology as per 2014 WHO guidelines.

We included high-grade (grade 2–3) serous carcinoma, and high-grade (grade 3) endometrioid cancers. In addition, we included historically used diagnoses, carcinosarcoma and carcinoma non-specified (NOS) that are no longer represented in current guidelines (2) Non-HGSC which included low-grade (grade 1) serous, endometrioid (grade 1–2), clear cell, mucinous, mixed and Brenner cancers.

Stage - Early (I–II) and advanced (III, IV and unable to stage) stage disease.

We used a chi-square test of independence and a significance level of 0.05 to provide evidence of a difference.

For completeness, we have provided symptom data in the PC and CD groups by randomisation group (MMS, USS and no screening) in the web tables.

This trial is registered with ISRCTN number 22488978; ClinicalTrials. gov number NCT00058032.

## 3 Results

At primary analysis, we were aware of a total of 1133 women with iEOC who had developed iEOC by Dec 31, 2014 [3]. Of them, 286 (181 MMS, 105 USS) were screen-detected (PC group) and 847 (118 MMS, 154 USS, 575 no screening) were clinically diagnosed (CD group). Women were predominantly White (98%; 1111/1133), 2% had a maternal history of ovarian cancer and 5% had a personal history of breast cancer. In both PC and CD groups, median age at recruitment was similar. The median age at diagnosis of iEOC was 66.8 years (IQR 62.3–71.6) in the CD and 68.7 years (IQR 63.2–73.7) in the CD group (*p* = 0.00009) (Table 1). Median age at diagnosis of HGSC was 67.0 years (IQR 63.0–71.6) in the PC and 69.0 years (IQR 64.0–73.8) in the CD group.

Of the women, 85% (964/1133) had HGSC (234 PC, 730 CD). The remaining 168 women with non-HGSC (51 PC, 117 CD) comprised of 37 with low-grade serous, 46 endometrioid, 48 clear cell, 34 mucinous, 2 mixed and 1 Brenner cancers.

### 3.1 Symptoms in women with invasive epithelial ovarian cancer (iEOC)

Among the PC women, 47% (133/286) reported symptoms when questioned, during clinical assessment (Table 2). Using the currently available options, 27% (77/286) of the PC women would have been detected using the modified GSI and 31% (88/286) by the NSG compared to 61% (515/847) and 64% (542/847) respectively of the CD women. In both SD and CD women, the median number of symptoms per women (2, IQR 1–3) was similar with similar proportions of symptomatic women reporting more than one symptom (56%, 75/133, SD; 59%, 447/761 CD). Abdominal symptoms were the most common. However, the symptom profile was different (Table 2, eTable 1). The.

### 3.2 Symptoms in women with HGSC

**Fig. 1:**
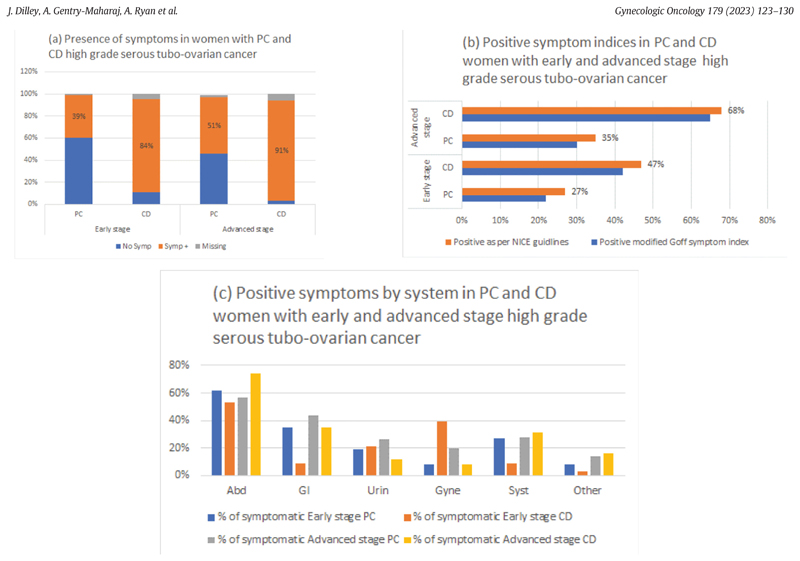
Symptoms in women with preclinical (PC) and clinically diagnosed (CD) early and advanced stage high grade serous tubo-ovarian cancer (a) Presence of symptoms (b) Positive symptom indices (c) Positive symptoms by system.

In the PC group, 39% (26/67) and 51% (86/167) respectively of women with early and advanced stage disease were symptomatic compared to 84% (80/95) and 91% (580/635) in the CD group. In the PC symptomatic women, significantly lower proportions were positive as per modified GSI (early stage 22%, 15/67; advanced stage 30%, 50/167) and NSG (early stage 27%, 18/67; advanced stage 35%, 58/167) compared to CD women (early stage 42%, 40/95; advanced stage 65%, 415/635 GSI, early stage 47%, 45/95; advanced stage 68%, 430/635 NSG) Most symptomatic women with HGSC had more than one symptom 60%,67/112 PC; 60%,398/660 CD) with abdominal symptoms most common, both in early (62%16/26, PC; 53%42/80, CD; *p* = 0.42) and advanced (57% 49/86, PC; 74%, 431/580, CD; *p* = 0.001) stage disease. However, the symptom profile was different in the symptomatic PC women with HGSC (Table 3, Fig. 1).

In symptomatic early stage HGSC, compared to the CD group, PC women reported more gastrointestinal (35%, 9/26 PC; 9%, 7/80; p = 0.001) and systemic (27%, 7/26 PC; 9%, 7/80; *p* = 0.017) symptoms (Table 3, Fig. 1). The gastrointestinal symptoms they reported were in the main change in bowel habits and dyspepsia and the reported systemic symptoms comprised mostly of lethargy /tiredness (eTable1). They reported fewer gynaecological (8%, 2/26 PC; 39%, 31/80; *p* = 0.003) symptoms, in particular vaginal bleeding (Table 3, eTable1).

In advanced stage disease, symptomatic PC women reported fewer abdominal (57%, 49/86 PC; 74%, 431/580; *p* = 0.001) and more gynaecological (20%, 17/86 PC; 8%, 48/580; p = 0.001) and urinary (26%, 22/86 PC; 12%, 71/580; p = 0.001) symptoms compared to the symptomatic CD group (Table 3, Fig. 1). Specifically, they reported less ‘increase in abdominal size/bloating’ and more urinary frequency and urgency compared to CD women. Additionally, fewer PC women reported weight loss and more reported lethargy and tiredness (eTable 1).

The differences were more pronounced in the MMS PC group where we have evidence of significant downstaging [11] (eTable 2).

### 3.3 Symptoms in women with non-HGSC

Majority had early stage disease (84 43/51 PC, 79%, 92/117 CD) in both groups. They had less symptoms than women with HGSC. Abdominal symptoms were most common and the symptom profile was similar in the PC and CD women (Table 4).

## 4 Discussion

To our knowledge, this is the first study of symptoms in women with screen-detected, pre-clinical iEOC. The finding that the median age at diagnosis of iEOC and HGSC in the PC group was two years younger than the CD attests to detection earlier in the natural history of the disease. Our findings suggest that half the women with iEOC experience symptoms upto two years prior to the cancer becoming clinically apparent. However, at the earlier point in the natural history, the symptom profile differs from that observed in women diagnosed clinically. While abdominal symptoms remain the most common, women with early stage preclinical HGSC report more gastrointestinal symptoms, mostly change in bowel habits and dyspepsia and more systemic symptoms, comprising largely of lethargy and tiredness. None reported a change in appetite/feeling full, a key ovarian cancer alert symptom and very few reported gynaecological symptoms, in particular vaginal bleeding. In advanced stage HGSC, women with preclinical disease report less abdominal and more gynaecological and urinary symptoms than those diagnosed clinically. The symptom profile of women with symptomatic preclinical non-HGSC was similar to that of women diagnosed clinically.

### 4.1 Strengths and weaknesses

Key strengths are that these analyses are nested within a multicentre randomised controlled trial of over 200,000 participants that includes over 670,000 annual screening episodes, and over 3 million women-years of follow-up. UKCTOCS has provided the first evidence of downstaging of HGSC with screening [11]. In the trial, linkage to multiple national registries and postal follow-up ensured completeness of ascertainment of cancers. iEOC diagnosis, stage and histotype was undertaken by independent outcome review.

A key weakness is that these are exploratory analysis and were not pre-specified outcomes of the trial. Although the trial has included 11 annual screens and shown evidence of downstaging of HGSC, the absolute number of screen detected women with early stage preclinical HGSC who were symptomatic were limited. However, they provide a unique opportunity to explore the symptom profile earlier in the natural history of HGSC. Data regarding symptoms was collected for women in UKCTOCS who did not have cancer as part of another study. However, this data has not been included in this analysis where the focus is on understanding the difference in symptoms between early stage preclinical and clinical HGSC. We hope to publish the baseline prevalence of these symptoms in due course to enable estimation of the positive predictive value how elevated some of these non-specific symptoms actually are in women with tubo-ovarian cancer. Finally, all women included the PC group were specifically questioned about symptoms as part of the trial clinical assessment process. This likely contributed to less missing data and more comprehensive reporting of symptoms compared to those who were clinically diagnosed.

### 4.2 Findings in the context of literature

The findings in the CD women in our study was consistent with that previously reported in case series [14–19] of clinically diagnosed ovarian cancer patients. In our study, 90% of CD women were symptomatic - 43% reported pelvic/abdominal pain or discomfort, 42% increase in abdominal size/bloating, 30% gastrointestinal symptoms and 21% urinary symptoms and 9% vaginal bleeding. The spread of symptoms aligns with that reported in a population based study [20] that like ours, only includes women diagnosed with iEOC. Of the 622 patients included, 52% reported abdominal pain, 41% distended abdomen, 39% bowel symptoms, 31% urinary symptoms and 10% vaginal bleeding. Like us, the latter also found variation in symptomatology based on histotype. Women with HGSC reported more symptoms than those with non-SHGSC. The findings confirms that the data sources and methods we used to extract symptom data were robust.

Although only half the women with preclinical HGSC were symptomatic, of those symptomatic, half had more than one symptom. This included half of those with symptomatic early stage preclinical HGSC. In the latter group, abdominal symptoms remained the most common with 38% reporting increase in abdominal size/bloating and one in five pelvic/abdominal pain or discomfort. Persistent abdominal bloating/distension is the symptom that is most commonly associated with ovarian cancer and has been widely adopted in awareness campaigns. Our findings lend further support to the importance this as a key ovarian cancer symptom. However, it is important we distinguish between ‘bloating’ which seems to be an early symptom and increase in abdominal size due to ascites and abdominal tumour which occurs with advanced disease. In keeping with this, the symptoms associated with increased mortality in patients with positive GSI and the NGS symptoms have been abdominal pain and feeling full/loss of appetite and not bloating [6]. In early stage disease, it is unclear as to the mechanism underlying the bloating that women experience. Recent studies have linked dysbiosis in the genital microbiota and inflammation to ovarian carcinogenesis [21] which raises the question as to whether this maybe the origin of these symptoms. In advanced stage preclinical HGSC, fewer women reported abdominal symptoms compared to those diagnosed clinically. This is likely related to the lower abdominal tumour volumes in screen-detected women with HGSC in the MMS group. Women with stage Ic-IV HGSC in the MMMS group had higher primary surgery rates and zero residual disease following debulking surgery compared to the no screening group [11].

Gastrointestinal symptoms were reported by 44% and 38% of PC and CD women respectively with advanced stage HGSC. What was notable was that a similar proportion (35%) of symptomatic women with early stage preclinical HGSC also reported these symptoms. This was significantly >9% reported by clinically diagnosed symptomatic early stage HGSC women in our study and the 5% reported in a retrospective chart review of 419 high-risk early-stage iEOC patients [22]. A key symptom included in this complex was dyspepsia which in our study included nausea and vomiting, indigestion and heartburn. In the recently reported Cancer Loyalty Card Study (CLOCS), the authors identified indigestion medication purchases as early as 13 months prior to diagnosis by women clinically diagnosed with stage III-IV ovarian cancer, predominantly high grade serous. No data was available for early stage HGSC as most of the stage I-II cases in CLOCS were borderline and non-HGSC. Of note, similar proportions of women with early stage non-HGSC also reported gastrointestinal symptoms. To date the view has been that gastrointestinal findings are an indicator of advanced disease with poor prognosis. Chase et al. found that in women with newly diagnosed ovarian cancer who were undergoing debulking surgery, those who had a claim within the past year for a gastrointestinal disorder were more likely to be unsuitable for primary cytoreduction surgery and require neoadjuvant chemotherapy [23]. Our findings of GI symptoms in early stage preclinical HGSC suggest that the lack of adequate numbers of early stage HGSC cases in such datasets might have skewed results. All the evidence to date suggests that iEOC should be considered early in the differential diagnosis of older women with gastrointestinal symptoms, in particular heartburn and dyspepsia. It would be prudent to enquire about other ovarian cancer symptoms in such situations. However, the prevalence of these symptoms must be set in context of how often women report these symptoms to a GP. In an earlier survey involving 51,007 postmeno-pausal women in UKCTOCS, 8% reported having discussed indigestion or heart burn with their GP during the preceding three months [24].

One in five symptomatic women with preclinical early stage HGSC reported lethargy and tiredness compared to one in ten of those with early stage CD. Of note, fatigue was reported by one-third of women with clinically diagnosed early stage iEOC [20] in the study by Lurie et al. We too found similar rates (29%) in an international survey of over 800 newly diagnosed ovarian cancer patients [19]. However we found that their primary care physicians only reported fatigue in 3% of the same cohort. Fatigue is under reported by clinicians. Poor recording in notes, possibly also contributed to our finding of fatigue in only 5% of clinically diagnosed women. There is much speculation about the role of biological factors in cancer-related fatigue with mounting support for the hypothesis that heightened inflammatory activity and proinflammatory cytokines contribute to cancer-related fatigue [25,26]. Again, as a symptom to aid differential diagnosis, this must be viewed in the context of 14% of 51,007 postmenopausal UKCTOCS women reporting that they had discussed tiredness, fatigue or lack of energy with their GP in the preceding three months [24].

Abnormal vaginal bleeding was a rare symptom in early stage pre-clinical HGSC. It is a symptom that in older postmenopausal women is rapidly reported and usually managed by a gynaecologist. As a result, those presenting with bleeding in early stage cancer are almost always clinically diagnosed. In keeping with this, in the trial, among women with symptomatic early stage clinically diagnosed HGSC, 31% (95%CI 22–42) reported abnormal bleeding. Overall 9% of women with early stage clinically diagnosed iEOC reported abnormal vaginal bleeding which is similar to the 12% reported by Lurie et al [20] for stage I-II iEOC and 13% by Chan et al [22] in high-risk early stage epithelial ovarian cancer.

Our finding of symptoms in preclinical patients as well as that of studies like CLOCS [27] put in question the timelines previously reported for patient intervals [16,19,28]. This probably reflects the poor recording on symptoms and their onset in medical records. It is likely that these limitations will be magnified with the current trend to ever shorter appointments and the perceived diminishing role of detailed symptom history given the extensive use of imaging. Recall bias of patients is also a contributary factor. There is need to elicit information on specific symptoms directly from patients to obtain accurate information for symptom research.

### 4.3 Implications

Our finding that half the women with screen detected invasive epithelial ovarian cancer reported symptoms when questioned provides renewed impetus to earlier diagnosis efforts in symptomatic women.

Our discovery that the symptom profile in early stage preclinical HGSC differs from that observed in clinical disease adds to the growing evidence that we should reconsider what constitutes an alert symptom for early disease. It is likely we need a more nuanced complex of key symptoms which is then evaluated and refined in a prospective trial.

It is important to note that it is unlikely that earlier diagnosis based on an improved understanding of symptoms will not impact on ovarian cancer mortality, given the results of UKCTOCS. To save lives will require a screening biomarker that detects the disease much earlier in its natural history before it is symptomatic.

## 5 Conclusions

The symptom profile earlier in the natural history of high grade serous tubo-ovarian cancer differs from that observed in women diagnosed clinically. While abdominal symptoms remain the most common, women report gastrointestinal symptoms– both change in bowel habits and dyspepsia as well as tiredness. It is important that new onset of more than one of these non-specific symptoms in postmenopausal women raises the possibility of invasive ovarian cancer.

## Supplementary Material

Supplementary data

## Figures and Tables

**Table 1:** Baseline characteristics of women with preclinical (PC) and clinically diagnosed (CD) invasive epithelial ovarian cancer.

Baseline characteristics	Women with preclinical invasive epithelial ovarian cancer	Women with clinically diagnosed invasive epithelial ovarian cancer
Number	286 (100)	847 (100)
Median (IQR) age atbaseline in years	62.5 (58.4–67.7)	62.9 (57.4–68)
Median (IQR)^a^ age at diagnosis in years	66.8 (62.3–71.6)	68.7 (63.2–73.7)
Ethnicity		
White	280 (97.9)	831 (98.1)
Non–white	3 (1)	7 (0.8)
Other	3 (1)	5 (0.6)
Missing	0 (0)	4 (0.5)
Hysterectomy	50 (17.5)	168 (19.8)
OCP use	130 (45.5)	422 (49.8)
Pregnancies <6 months	0 (0–1)	0 (0–1)
Children	2 (2–3)	2 (1–3)
Personal history of breast cancer	9 (3.1)	42 (5)
Maternal history of ovarian cancer	6 (2.1)	16 (1.9)
Maternal history of breast cancer	24 (8.4)	57 (6.7)

Data are n (%) or median (IQR).IQR, interquartile range.

a Lower age compared to clinically diagnosed is an approximate measure of lead time of screening.

**Table 2:** Symptom profile in women with preclinical (PC) and clinically diagnosed (CD) invasive epithelial ovarian cancer - overall and by stage.

Characteristics	Overall				Early stage^a^				Advanced stage^b^		
	PC	CD	*p* value^c^		PC	CD	p value^c^		PC	CD	p value^c^
Overall	286 (100)	847 (100)			110 (100)	187 (100)			176 (100)	660 (100)	
Asymptomatic	147 (51)	29 (3)			63 (57)	13 (7)			84 (48)	16 (2)	
Symptomatic	133 (47)	761 (90)			45 (41)	159 (85)			88 (50)	602 (91)	
Missing	6 (2)	57 (7)			2 (2)	15 (8)			4 (2)	42 (6)	
Positive on modified Goff Symptom index	77 (27)	515 (61)	0.000		26 (24)	86 (46)	0.009		51 (29)	429 (65)	0.000
Positive as per NICE guidelines	88 (31)	542 (64)	0.000		29 (26)	97 (52)	0.008		59 (34)	445 (67)	0.000
Symptomatic women	133 (100)	761 (100)			45 (100)	159 (100)			88 (100)	602 (100)	
Median (IQR) number of symptoms	2 (1–3)	2 (1–3)	0.872		1 (1–2)	1 (1–2)	0.725		2 (1–3)	2 (1–3)	0.857
More than one symptom	75 (56)	447 (59)	0.612		20 (44)	66 (42)	0.725		55 (63)	381 (63)	0.886
Abdominal symptoms	77 (58)	537 (71)	0.004		27 (60)	94 (59)	0.915		50 (57)	443 (74)	0.001
Gastrointestinal symptoms	54 (41)	230 (30)	0.018		15 (33)	20 (13)	0.001		39 (44)	210 (35)	0.085
Urinary symptoms	33 (25)	104 (14)	0.001		11 (24)	32 (20)	0.531		22 (25)	72 (12)	0.001
Gynaecological symptoms	21 (16)	98 (13)	0.362		4 (9)	46 (29)	0.006		17 (19)	52 (9)	0.002
Systemic symptoms	34 (26)	204 (27)	0.765		10 (22)	18 (11)	0.061		24 (27)	186 (31)	0.490
Other symptoms	15 (11)	105 (14)	0.432		2 (4)	9 (6)	0.750		13 (15)	96 (16)	0.778

Data are n (%) or median (IQR). IQR = interquartile range.

a Stage I and II.

b Stage III/IV/unable to stage.

c PC vs CD.

**Table 3:** Symptom profile in women with preclinical (PC) and clinically diagnosed (CD) high-grade serous tubo-ovarian cancer (HGSC) - overall and by stage.

Characteristics	Overall				Early stage^a^				Advanced stage^b^		
	PC	CD	*p* value^c^		PC	CD	p value^c^		PC	CD	p value^c^
Overall	234 (100)	730 (100)			67 (100)	95 (100)			167 (100)	635 (100)	
Asymptomatic	117 (50)	26 (4)			40 (60)	10 (11)			77 (46)	16 (3)	
Symptomatic	112 (48)	660 (90)			26 (39)	80 (84)			86 (51)	580 (91)	
Missing	5 (2)	44 (6)			1 (1)	5 (5)			4 (2)	39 (6)	
Positive GSI^d^	65 (28)	455 (62)	0.000		15 (22)	40 (42)	0.000		50 (30)	415 (65)	0.000
Positive as per NICE guidelines	76 (32)	475 (65)	0.000		18 (27)	45 (47)	0.000		58 (35)	430 (68)	0.000
Symptomatic women	112 (100)	660 (100)			26 (100)	80 (100)			86 (100)	580 (100)	
Median (IQR) number of symptoms	2 (1–3)	2 (1–3)	0.974		2 (1–2)	1 (1–2)	0.213		2 (1–3)	2 (1–3)	0.998
More than one symptom	67 (60)	398 (60)	0.923		13 (50)	29 (36)	0.213		54 (63)	369 (64)	0.881
Abdominal symptoms	65 (58)	473 (72)	0.004		16 (62)	42 (53)	0.421		49 (57)	431 (74)	0.001
Gastrointestinal symptoms	47 (42)	211 (32)	0.038		9 (35)	7 (9)	0.001		38 (44)	204 (35)	0.105
Urinary symptoms	27 (24)	88 (13)	0.003		5 (19)	17 (21)	0.825		22 (26)	71 (12)	0.001
Gynaecological symptoms	19 (17)	79 (12)	0.142		2 (8)	31 (39)	0.003		17 (20)	48 (8)	0.001
Systemic symptoms	31 (28)	187 (28)	0.887		7 (27)	7 (9)	0.017		24 (28)	180 (31)	0.557
Other symptoms	14 (13)	94 (14)	0.623		2 (8)	2 (3)	0.227		12 (14)	92 (16)	0.649

Data are n (%) or median (IQR). IQR = Interquartile Range.

a Stage I and II.

b Stage III/IV/unable to stage.

c PC vs CD.

d Modified Goff Symptom index.

**Table 4:** Symptom profile in women with preclinical (PC) and clinically diagnosed (CD) invasive non high-grade serous ovarian cancer (non-HGSC) - overall and by stage.

Characteristics	Overall				Early stage^a^				Advanced stage^b^		
	PC	CD	*p* value^c^		PC	CD	p value^c^		PC	CD	p value^c^
Overall	51 (100)	117 (100)			43 (100)	92 (100)			8 (100)	25 (100)	
Asymptomatic	30 (59)	3 (3)			23 (53)	3 (3)			7 (88)	0 (0)	
Symptomatic	20 (39)	101 (86)			19 (44)	79 (86)			1 (13)	22 (88)	
Missing	1 (2)	13 (11)			1 (2)	10 (11)			0 (0)	3 (12)	
Positive on modified Goff Symptom index	12 (24)	60 (51)	0.001		11 (26)	46 (50)	0.007		1 (13)	14 (56)	0.032
Positive as per NICE guidelines	12 (24)	67 (57)	0.000		11 (26)	52 (57)	0.001		1 (13)	15 (60)	0.019
Symptomatic women	20 (100)	101 (100)			19 (100)	79 (100)			1 (100)	22 (100)	
Median (IQR) number of symptoms	1 (1–2)	1 (1–2)	0.268		1 (1–2)	1 (1–2)	0.432		1 (1–1)	2 (1–2)	0.639
More than one symptom	7 (35)	49 (49)	0.268		7 (37)	37 (47)	0.432		0 (0)	12 (55)	0.286
Abdominal symptoms	12 (60)	64 (63)	0.804		11 (58)	52 (66)	0.597		1 (100)	12 (55)	1.000
Gastrointestinal symptoms	6 (30)	19 (19)	0.363		6 (32)	13 (16)	0.193		0 (0)	6 (27)	1.000
Urinary symptoms	6 (30)	16 (16)	0.200		6 (32)	15 (19)	0.230		0 (0)	1 (5)	1.000
Gynaecological symptoms	2 (10)	19 (19)	0.521		2 (11)	15 (19)	0.513		0 (0)	4 (18)	1.000
Systemic symptoms	3 (15)	17 (17)	1.000		3 (16)	11 (14)	1.000		0 (0)	6 (27)	1.000
Other symptoms	0 (0)	11 (11)	0.208		0 (0)	7 (9)	0.340		0 (0)	4 (18)	1.000

Data are n (%) or median (IQR). IQR = Interquartile Range.

a Stage I and II.

b Stage III/IV/unable to stage.

c PC vs CD.

## Data Availability

The trial protocol is available on the study website. The individual participant data that underlie the results reported in this Article, after de-identification, will be available beginning 12 months after publication. A data dictionary defining each field in the set will be made available. Researchers will need to state the aims of any analyses and provide a methodologically sound proposal. Proposals should be directed to u.menon@ucl.ac.uk. Data requestors will need to sign a data access agreement and in keeping with patient consent for secondary use, obtain ethical approval for any new analyses. Following all necessary approvals and mandatory training required for access to UKCTOCS data, the researchers will be given access to the data which is housed within the UCL Data Safe Haven.

## Highlights

- High alert ovarian cancer symptoms based on reports by clinically diagnosed women are associated with poorer outcomes.
- In UKCTOCS, we explore prospectively preclinical symptoms in women detected by screening earlier in the natural history.
- In high grade serous ovarian cancer, the symptom profile was different between women with preclinical and clinical disease.
- Women with preclinical early stage high grade serous ovarian cancer reported more gastrointestinal and systemic symptoms.
- Key symptoms were change in bowel habits, dyspepsia and tiredness.
